# Current perspectives on endovascular therapy for large core ischemic stroke

**DOI:** 10.1016/j.neurot.2025.e00622

**Published:** 2025-06-16

**Authors:** Chloe A. Mutimer, Bruce C.V. Campbell

**Affiliations:** Department of Medicine and Neurology, Melbourne Brain Centre at the Royal Melbourne Hospital, University of Melbourne, Parkville, Victoria, Australia

**Keywords:** Ischemic stroke, Endovascular therapy, Large ischemic core, Low ASPECTS

## Abstract

Historically, patients with ischemic stroke and an extensive region of irreversibly injured ischemic core were excluded from endovascular thrombectomy trials due to concerns about limited benefit and high procedural risk. This has fundamentally changed with the publication of five strongly positive randomized controlled trials in this group of patients since 2022 and a sixth trial that showed consistent trends and was positive in per protocol analysis and long-term follow-up. This narrative review summarizes the key findings of these trials, including imaging selection criteria, functional and safety outcomes, and long-term benefits. Across trials, thrombectomy consistently improved functional outcomes at 3-months and 12-months. Absolute functional independence (modified Rankin Scale score 0–2) rates were lower than the trials that enrolled patients with smaller ischemic core volumes, but still significantly favored thrombectomy. Safety outcomes demonstrated a reduction in mortality with EVT and no significant increase in rates of symptomatic intracerebral hemorrhage. While guidelines are being updated to include large core thrombectomy, real-world decision-making remains complex, requiring careful consideration of patient-specific factors, including functional status, infarct location, and patient preferences. Future research should focus on exploring adjunctive therapies and accelerated systems of care to further improve outcomes in this patient population.

## Introduction

The “core” in ischemic stroke has typically been defined as hypoperfused brain tissue that is irreversibly injured, while the “penumbra” represents hypoperfused but potentially salvageable tissue [1]. The pivotal trials of endovascular thrombectomy for acute ischemic stroke due to large vessel occlusion, initially within 6 ​h from symptom onset, and subsequently up to 24 ​h, largely excluded patients with large ischemic core based on imaging-based constructs [[2], [3], [4], [5], [6], [7]]. Patients were either excluded based on their Alberta Stroke Program Early CT Score (ASPECTS) ​< ​6 in the early timeframe (<6 ​h from onset) or >50 ​ml (DAWN) or >70 ​ml (DEFUSE3) core up to 24 ​h, defined using CT-perfusion or diffusion MRI [8,9]. Despite patients with large ischemic core representing ∼20 ​% of those with large vessel occlusions, the rationale for excluding this group was the perceived futility and safety when a large area of brain was already injured, and needs to be viewed in the historical context of the need to prove a therapy that had 3 neutral trials published in 2013 [[10], [11], [12], [13]]. The more recent publication of five positive randomized controlled trials (and a 6th neutral trial) of endovascular thrombectomy versus medical management for large core ischemic stroke due to large vessel occlusion has led to a paradigm shift and change in practice in the management of these patients [[14], [15], [16], [17], [18], [19]]. In this review, we will summarize the evidence from these randomized controlled trials, including clinical and imaging selection, and discuss current practice and future directions.

## Large Core Thrombectomy Randomized Controlled Trials

Since 2022, there have been five positive randomized controlled trials of endovascular thrombectomy compared to medical management for large core ischemic stroke (Recovery by Endovascular Salvage for Cerebral Ultra-Acute Embolism–Japan Large Ischemic Core Trial [RESCUE-Japan LIMIT], Endovascular Therapy in Acute Anterior Circulation Large Vessel Occlusive Patients with a Large Infarct Core [ANGEL-ASPECT], A Randomized Controlled Trial to Optimize Patient's Selection for Endovascular Treatment in Acute Ischemic Stroke [SELECT2], Efficacy, Safety of Thrombectomy in Stroke With Extended Lesion and Extended Time Window [TENSION] and LArge Stroke Therapy Evaluation [LASTE]) and one neutral trial with directionally similar results (Thrombectomy for Emergent Salvage of Large Anterior Circulation Ischemic Stroke [TESLA]). All trials included patients with anterior circulation large vessel occlusion (distal internal carotid artery or the first portion of the middle cerebral artery; with the exception of LASTE where proximal M2 occlusions were also included). Patients were largely under 80 or 85 years old and had excellent pre-morbid function (modified Rankin Scale [mRS] 0 or 1), with the exception of TENSION in which patients with pre-stroke mRS 2 were also included. All trials had slightly differing clinical and imaging inclusion criteria, as outlined in Table 1. The primary endpoint in 4 of the 6 trials was ordinal analysis of mRS distribution at 90-days, while in RESCUE-Japan LIMIT the primary endpoint was independent ambulation (mRS 0–3) at 90-days and in TESLA was utility-weighted mRS at 90-days.

**Table 1:** Summary of clinical and imaging inclusion criteria in the six randomized controlled trials of endovascular thrombectomy for large core ischemic stroke.

Study, year	Region(s), sample size (n)	Pertinent inclusion criteria			Imaging selection		
		Age	Baseline NIHSS	Time from onset	ASPECTS	CT-perfusion	MRI
**RESCUE-Japan LIMIT, 2022** [14]	Japan; 203	≥18	≥6	≤6 ​h, or ≤24 ​h if met MRI criteria	3–5	N/A	DWI-ASPECTS 3-5
**ANGEL-ASPECT, 2023** [15]	China; 456	18–80	6–30	≤24 ​h	3-5 with no limitation in volume; 0–2, or >5 with core volume 70–100 ​ml	N/A	N/A
**SELECT2, 2023** [16]	North America, Australia, New Zealand, Europe; 352	18–85	≥6	≤24 ​h	3–5	≥50 ​ml (CBF <30 ​%)	≥50 ​ml (ADC <620 ​× ​10^−6^mm^2^
**TENSION, 2023** [17]	Europe, Canada; 253	≥18	<26	≤11 ​h	3–5	N/A	DWI-ASPECTS 3-5
**LASTE, 2024** [18]	Europe; 333	≥18	≥6	≤6.5 ​h or ≤24 ​h if met MRI criteria	≤5 if ​< ​80yo 4-5 if ​≥ ​80yo	N/A	No FLAIR-DWI correlate
**TESLA, 2024** [19]	North American, Europe; 302	18–85	>6	≤24 ​h	2–5	N/A	N/A

### Functional Outcomes at 90-days

**Fig. 1:**
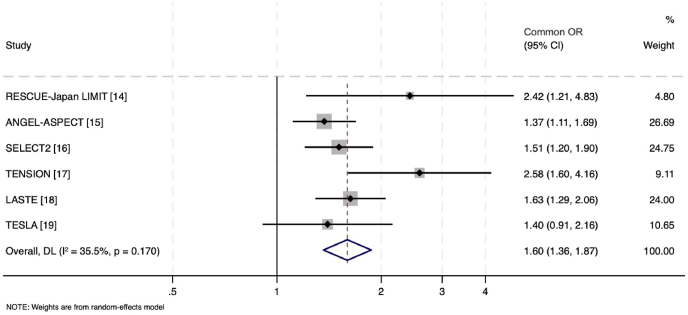
Study-level meta-analysis of trials in large core thrombectomy for the outcome ordinal mRS at 90-days.

Five trials (ANGEL-ASPECT, RESCUE-Japan LIMIT, SELECT2, TENSION and LASTE) showed improved functional outcomes with endovascular thrombectomy when compared to medical management, with major outcomes summarized in Table 2. Ordinal mRS outcomes using study-level data from all 6 trials is shown in Fig. 1.

**Table 2:** Summary of primary and secondary outcomes, including safety outcomes, in the six randomized controlled trials of endovascular thrombectomy for large core ischemic stroke.

Study	Ordinal mRS at 90-days	Functional independence (mRS 0–2) at 90-days	Independent ambulation (mRS 0–3) at 90-days	Mortality	Symptomatic intracranial hemorrhage	Decompressive hemicraniectomy
**RESCUE-Japan LIMIT, 2022** [14]	OR 2.42 (95%CI 1.76–7.00)	RR 1.79 (95%CI 0.78–4.07)	RR 2.43 (95%CI 1.35–4.37)	RR 0.77 (95%CI 0.44–1.32)	RR 1.84 (95%CI 0.64–5.29)	0.73 (95%CI 0.34–1.56)^a^
**ANGEL-ASPECT, 2023** [15]	OR 1.37 (95 ​% CI 1.11–1.69)	RR 2.62 (95%CI 1.69–4.06)	RR 1.50 (95%CI 1.17–1.91)	HR 1.00 (95%CI 0.65–3.84)	RR 2.07 (95%CI 0.79–5.41)	RR 1.92 (95%CI 0.78–4.73)
**SELECT2, 2023** [16]	OR 1.51 (95%CI 1.20–1.89)	RR 2.97 (95%CI 1.60–5.51)	RR 2.06 (95%CI 1.43–2.96)	RR 0.91 (95%CI 0.71–1.18)	RR 0.49 (95%CI 0.04–5.36)	NR
**TENSION, 2023** [17]	OR 2.58 (95%CI 1.60–4.15)	aOR 7.16 (95%CI 2.12–24.21)	aOR 2.84 (95%CI 1.48–5.47)	aHR 0.67 (95%CI 0.46–0.98)	5 ​% EVT vs. 5 ​% MM (p ​= ​1.00)	8.8 ​% EVT vss. 7.0 ​% MM (p ​= ​0.65)
**LASTE, 2024** [18]	OR 1.63 (95%CI 1.29–2.06)	aRR 3.26 (95%CI 1.67–8.46)	aRR 2.67 (95%CI 1.79–4.41)	aRR 0.65 (95%CI 0.50–0.84)	aRR 1.73 (95%CI 0.78–4.68)	NR
**TESLA, 2024** [19]	OR 1.40 (95%CI 0.91–2.16)	RR 1.64 (95%CI 0.86–3.12)	RR 1.50 (95%CI 1.00–2.26)	RR 1.06 (95%CI 0.77–1.45)	RR 2.96 (95%CI 0.61–2.96)	RR 1.48 (95%CI 0.91–2.42)

a Within 7 days.

**Fig. 2:**
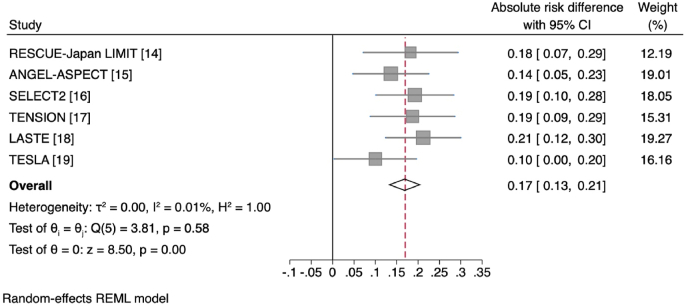
Forest plot of absolute risk differences in good functional outcome (mRS 0–3 ​at 90 days) across study-level data for individual trials.

**Fig. 3:**
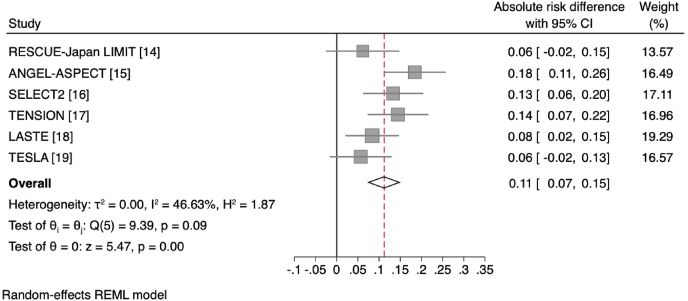
Forest plot of absolute risk differences in excellent functional outcome (mRS 0–2 ​at 90 days) across study-level data for individual trials.

Endovascular thrombectomy in all trials showed benefit for independent ambulation (defined as mRS 0–3). When pooling patient results from all trials in a study-level meta-analysis, the number needed to treat was 5.9 (95%CI 4.8–7.7) for good functional outcome (mRS 0–3; pooled absolute risk difference 16.9 ​% [95%CI 13.0–20.7 ​%], Fig. 2). Rates of functional independence (mRS 0–2) were also higher in the endovascular thrombectomy group in all trials, although the absolute proportions were low. When pooling results from all trials in a study-level meta-analysis, the number needed to treat was 9.0 (95%CI 6.6–14.0) for excellent functional outcome (pooled absolute risk difference 11.2 ​% [95%CI 7.2–15.2 ​%], Fig. 3). Trials varied in the median ischemic core volume of patients included, and rates of functional independence varied accordingly, with the highest rates in ANGEL-ASPECT (mRS 0–2: 30 ​% in thrombectomy group, median core volume 62 ​ml) and lowest rates in LASTE (mRS 0–2: 13.3 ​%, median core volume 135 ​ml) [15,18]. Importantly, from a patient perspective, endovascular thrombectomy reduced the proportion of patients who were highly dependent, requiring constant nursing care (mRS 5). This was a consistent finding across trials, with pooled rates of 12.2 ​% in the endovascular thrombectomy group and 20.6 ​% in the medical management group. However, it is important to place this in context of the strict limits on premorbid disability and co-morbidity imposed by trial eligibility criteria. Many patients encountered in clinical practice fall outside these criteria and may have greater risk of survival with severe disability.

### Safety Outcomes

In all trials, mortality and symptomatic intracerebral hemorrhage were major safety outcomes, with a summary of these results shown in Table 2. Mortality was numerically lower with EVT in all trials and significantly so in LASTE and TENSION. Mortality was highest in LASTE, which had the largest median core volume: 36.1 ​% of patients in the endovascular thrombectomy group and 55.5 ​% in the medical management group had died by 90 days. In study-level meta-analysis, there was a statistically significant reduction in mortality [20]. In contrast to data in patients with smaller core, EVT is therefore not just disability-reducing but life-saving in this group of patients.

Given endovascular thrombectomy leads to reperfusion of a large volume of infarcted tissue, rates of intracerebral hemorrhage were expected to be higher, compared to patients not reperfused (medical management) or those with smaller core volumes [21]. While symptomatic intracerebral hemorrhage rates were indeed numerically higher in most trials in the thrombectomy group (except SELECT2), there were no statistically significant differences in rates of symptomatic intracerebral hemorrhage between endovascular thrombectomy and medical management in individual trials. Individual patient data meta-analysis is pending and will be important to harmonize definitions of symptomatic hemorrhage.

Rates of decompressive hemicraniectomy were reported in 5 of the 6 trials (RESCUE-Japan LIMIT, ANGEL-ASPECT, TENSION, LASTE, TESLA), and did not differ between treatment groups.

### Long-term Outcomes

One-year outcomes have been published for SELECT2 and TENSION and presented for TESLA [[22], [23], [24]]. The beneficial effect of endovascular thrombectomy was sustained at 12-months in ordinal mRS assessment (SELECT2: aOR 1.53 [95%CI 1.23–1.90], TENSION: aOR 2.39 [95%CI 1.47–3.90]) and EVT benefit in the TESLA trial became statistically significant with longer term follow-up. Mortality rates at 12-months approached 50 ​% in both SELECT2 and TENSION but, reassuringly, rates of severely dependent patients (mRS 5) were not increased in the thrombectomy group. These studies also explored patient-reported quality of life outcomes, through the Neuro-QOL (physical, mental, cognitive and social effects of neurological conditions), EQ-5D (mobility, self-care, usual activities, pain or discomfort, and anxiety or depression), and PROMIS-10 (physical health and activities, fatigue and pain). In SELECT2, patients in the thrombectomy group had significantly better mobility, social health (including community access) and cognitive scores, and lower depression scores [22]. The follow-up outcomes in TENSION were similar with improved quality of life reflected as higher values on the EQ-5D index [23].

## The Role of Imaging and Integration With Clinical Factors

All trials variably used non-contrast CT, CT-perfusion or diffusion MRI for enrollment, with varying definitions of large ischemic core (as highlighted in Table 1). Ischemic core is represented differently by each modality: tissue hypodensity due to increased water uptake on non-contrast CT, reduced cerebral blood flow (compared to the contralateral side) on CT-perfusion imaging and diffusion restriction of water molecules due to cytotoxic edema on MRI [25,26]. Additionally, the way in which core can be quantified also differs. ASPECTS is a semiquantitative measure of core on non-contrast CT and MRI, whereas CT-perfusion processing provides a quantitative estimate of volume [27].

Given this, the benefit of endovascular thrombectomy compared to medical management using different imaging modalities was assessed in the SELECT2 trial where non-contrast CT and CT-perfusion (or, in a few cases, MR perfusion-diffusion imaging) was available for all patients [28]. In this study, point estimates favored endovascular thrombectomy in all subgroups, despite varying definitions and thresholds of large ischemic core (e.g. ≥70 ​ml, ≥100 ​ml, ≥150 ​ml) and acquisition techniques. Absolute rates of functional independence decreased significantly in patients receiving EVT, aRR 0.89 [95%CI 0.84–0.85] per 10 ​ml increase in core, and independent ambulation aRR 0.91 [95%CI 1.02–1.08] per 10 ​ml increase in core, but were correspondingly reduced in the medical management arm [28]. There was no clear upper limit of core volume beyond which EVT was not beneficial. Similar results were observed in the LASTE trial that had no upper limit on core for patients aged <80 but only enrolled patients <6.5 ​h from onset, predominantly using MRI. The clear benefit in patients with >150 ​mL core volume or with ASPECTS 0–2 in LASTE raises the potential that neither ASPECTS nor core volume require consideration in the early time window.

Some have interpreted this as reason to skip imaging altogether and proceed directly to the angiography suite in patients with severe clinical stroke syndromes. Flat panel CT can exclude hemorrhage and, in the presence of severe deficits, it is likely (with ∼80 ​% positive predictive value when NIHSS ≥10 and hemorrhage is excluded) that there is a large vessel occlusion [29]. Undoubtedly this direct-to-angiography suite approach will save time and patients with large ischemic core have the most to gain from faster treatment. However, the resource implications and potential delays to treatment for the majority of patients, who have non-LVO stroke and intracerebral hemorrhage, require further study and economic evaluation [30]. There also needs to be a clear distinction between true direct-to-angiography suite from the field (with no prior imaging) and skipping re-imaging of patients transferred from another hospital, the latter forming the predominant group in prior direct-to-angiography suite studies and driving the observed benefit [29]. Re-imaging patients who have been transferred because they are already known to have a target for EVT, and have been considered suitable candidates on clinical grounds, is increasingly difficult to justify when large core trials indicate such broad benefit. It is also critical that we retain perspective on the differences between patients eligible for randomized trials and the overall group of patients with large core stroke presenting to hospital, many of whom have advanced age, frailty and comorbidity. In such patients, in whom the potential benefit of EVT on quality of life is less certain, enhanced prognostic information from imaging may retain value in decision-making. A favorable imaging profile, with absence of core in critically eloquent regions, increases the likelihood that rapid reperfusion could return the patient to a similar level of function. In contrast, a large core may make what was previously an acceptable quality of life unacceptable. It is also important to recognize the value of transitions in mRS other than those that reach 0–2. Achieving independent ambulation (mRS 3) and perhaps even mRS 4 rather than mRS 5–6 is weighted more strongly by patients in health utility ratings. Achieving patient-centered decision-making in a time critical emergency is a challenging but worthwhile endeavor and imaging can be highly informative for prognosis.

If we accept that imaging retains an important role in diagnosis and prognostication for the real-world patients encountered outside clinical trials, then the optimal modality is that which can provide the most accurate information with the minimum delay. In the early timeframe (<3 ​h), non-contrast CT-hypodensity underestimates core compared to CT-perfusion or diffusion MRI, as tissue water uptake that causes hypodensity takes time to develop [28]. In contrast, CT-perfusion imaging often underestimates core in the later time window when collateral improvement or partial reperfusion can raise the cerebral blood flow above thresholds used to define core but too late to salvage brain tissue [28]. The best information therefore comes from combining these modalities. The “composite estimated core volume” (defined in SELECT2 as the larger of the two volumes on either CT-perfusion or non-contrast CT), was found to be most strongly associated with outcomes when compared to core volume defined by individual imaging modalities. In practice, automated methods to segment non-contrast CT hypodensity are rapidly improving and have the potential to be combined with CT-perfusion estimates in real time with future software advances. There is also preliminary evidence that the severity of the CT hypodensity, reflecting blood-brain-barrier failure, influences the treatment benefit of EVT and may lead to accelerated edema post-reperfusion [31].

MRI was the predominant imaging modality in LASTE (used in 83.6 ​% of patients), which enrolled individuals in an early timeframe (<6.5 ​h from symptom onset) [18]. Patients with unknown onset time could also be included if they presented within 24 ​h of last known well and demonstrated a diffusion-weighted imaging (DWI) lesion without a corresponding fluid-attenuated inversion recovery (FLAIR) lesion, suggesting onset within 4.5 ​h [32]. The use of DWI-FLAIR mismatch in this context improved the ability to select patients in the early timeframe while avoiding infarct underestimation, a limitation of CT, and may be of utility in centers with immediate access to MRI. Although MRI is more accurate than CT or CT-perfusion in core volume estimation, immediate access is much less widely available, meaning that CT is the most practical modality to assess ischemic core in most systems worldwide.

The discussion of core volume and the desire of some clinicians to set an upper limit on the volume, beyond which patients do not benefit, is predicated on a dichotomous view of infarcted versus salvaged brain tissue. The reality is more complicated. There is a gradient of injury within the ischemic zone, apparent on follow-up scans, which ranges from mild gliosis to complete liquefaction with more subtle changes evident using advanced MRI techniques such as diffusion tensor imaging [33]. In the pivotal trials of thrombectomy, patients with the same total follow-up infarct volume had better outcomes if they had received EVT and some of the positive large core trials achieved functional benefit without demonstrating a reduction in infarct volume [34]. This supports the concept that a reduction of ischemic injury intensity within the core may be as important as reductions in territorial growth of the infarct. It is unlikely that any single core volume threshold will define patients who will not benefit from thrombectomy. The volume but also location of core needs to be considered alongside individual patient factors – frailty, co-morbidity, likely ability to engage in intensive rehabilitation over months and attitudes to disability.

Another element of imaging that was central to trials that extended the time window for EVT and IV thrombolytic is perfusion mismatch. In both SELECT2 and ANGEL-ASPECT, patients who did not meet traditional definitions of mismatch (as an estimate of the salvageable ischemic penumbra) still appeared to benefit from EVT. There were relatively few patients without mismatch, partly due to underestimation of ischemic core by CTP in the late time window. However, even accounting for this using composite core volume in SELECT2, there was no evidence that patients without mismatch should be excluded from EVT [4,9]. It was also shown that, as mismatch volume increased, the probability of functional independence and independent ambulation increased for patients in the thrombectomy group but decreased in patients receiving medical management only [28]. It may be that the threshold required to meet mismatch definitions when the core volume is large (i.e. >20 ​mL when core is 100 ​mL to meet mismatch ratio >1.2) is larger than the volume of tissue required to be saved to improve outcome. Alternatively, reduction of ischemic injury severity within the core, as discussed above, may be relevant.

## Current Practice, Guidelines and Considerations

Thrombectomy for large core ischemic stroke was incorporated into the Australian and New Zealand Stroke Foundation Living Guidelines in July 2023: “For patients with a disabling clinical deficit due to ischemic stroke caused by a large vessel occlusion in the internal carotid artery, proximal middle cerebral artery (M1 and proximal or dominant M2 segments), basilar artery occlusion, or with tandem occlusion of both the cervical carotid and intracranial large arteries, endovascular therapy should be undertaken when the procedure can be commenced within 24 ​h of stroke onset, taking into account individual patient factors. Such factors include: extent and location of brain injury, pre-morbid function, frailty, comorbidities, and patient's and/or family's wishes.” [35] The European, American and Chinese guidelines are yet to be updated in relation to large core thrombectomy, although there has been a recent scientific advisory statement from the American Heart Association [36].

### Time From Symptom Onset

As has long been the paradigm in stroke care, time to treatment is strongly associated with outcomes and this is particularly true in fast progressing patients with large ischemic core. This is an especially important consideration in patients who do not present to thrombectomy-capable centers. A pre-specified analysis of the SELECT2 trial compared transferred patients to those who presented to thrombectomy-capable centers [37]. The median transfer time in this cohort was almost 3 ​h (176 ​min, interquartile range 128–231 ​min). It was shown that the benefit of endovascular thrombectomy for transferred patients was consistent for improvement in mRS, as well as functional independence and independent ambulation. However, treatment effect estimates were lower for transferred patients, with numerically higher rates of independent ambulation in the thrombectomy group with transfer time <3 ​h: 45 ​% vs. ≥3 ​h: 36 ​%. Similarly, functional independence in the thrombectomy group was numerically higher with transfer time <3 ​h: 25 ​% vs. ≥3 ​h: 18 ​%). Time to treatment therefore remains relevant and clinical and radiological deterioration during transport risks a futile transfer. Deterioration in ASPECTS was seen in transferred patients in the SELECT2 cohort, with a median loss of 1 point in patients randomized to thrombectomy. This was associated with numerically worse functional outcomes among patients who underwent thrombectomy (aOR 0.89; 95 ​% CI, 0.77–1.02 per 1-ASPECTS point loss). These factors should be considered in patients for whom reperfusion may be delayed but, as outlined, benefit of thrombectomy versus medical management is still evident. Further advancement in reducing stroke evolution, such as brain cytoprotectants, will be important to improving outcomes in this population.

## Patient Wishes and Withdrawal of Care Decisions

Pre-existing beliefs from patients and their families may impact the decision to offer thrombectomy. Factors already discussed, such as age and frailty, and location of infarct may be considered, as well as pre-existing beliefs about disability. Conveying long-term outcomes is important as patients with large ischemic core are often initially more clinically unwell and do not experience the same early neurological recovery seen after thrombectomy in the smaller core cohort. If the decision is made to proceed with endovascular thrombectomy, early withdrawal of support should be avoided, and families should be counseled about the potential stormy early course.

## Cost-effectiveness

From a societal perspective, it is important to understand the cost-effectiveness of the expansion of thrombectomy criteria to the patients with large ischemic core strokes. Despite being a resource-intensive therapy with high upfront costs, endovascular thrombectomy in large core ischemic stroke has been shown to be cost effective over a lifetime. A cost-effectiveness analysis of SELECT2 demonstrated that patients receiving endovascular thrombectomy had an average gain to 1.24 quality-adjusted life years which equated to an average reduction in healthcare costs of between $10,000 and 30,000 depending on region (patients from USA, Canada, Australia and Spain) [38]. Through sensitivity analyses of the other large core trials, cost-effectiveness of thrombectomy has also been demonstrated, despite inclusion of patients from different regions, using differing imaging modalities and definition of core volumes. However, patients with the largest cores in the LASTE trial had lower cost-effectiveness, chiefly because survival with moderate disability incurs greater cost than death. Cost-effectiveness has also been directly demonstrated in the Japanese setting [39].

## Future Directions

Subgroup analyses of thrombectomy for large core ischemic stroke has shown benefit in most patients. However, further investigation into specific clinical or imaging factors to help identify patients who do not benefit from thrombectomy will be important in applying these data to individual patients. This includes advancing age as previously discussed, as well as the degree of hypoperfused tissue and relative eloquence of the affected brain.

Despite improved outcomes with thrombectomy, a substantial proportion of patients with both small and large core ischemic strokes do not regain functional independence after successful macrovascular reperfusion. This has been attributed to several factors, but one proposed rationale is persistent microvascular hypoperfusion, termed “no-reflow” [40]. These regions have been found to exhibit evidence of microvascular pathology with pericyte swelling and entrapped erythrocytes, leukocytes and platelets [41]. The ability to modulate no-reflow, as well as treat non-retrievable thrombus, is being investigated in a number of trials of adjuvant intra-arterial thrombolytics. Initial results have indicated an improvement in mRS 0–1 but not in other mRS transitions. One ongoing trial includes patients with large ischemic core (ASPECTS 3–5, EXTEND AGNES NCT05892510).

A number of different brain cytoprotective agents have been investigated, including nerinetide in the neutral ESCAPE-NEXT trial, and ApTOLL in a phase 2 trial [42,43]. Edema may be exacerbated by reperfusion in patients with large core and is one reason for increased early neurological deterioration observed in some of the large core trials [16]. Agents such as intravenous glibenclamide may have a role in improving the safety of endovascular therapy through preserving blood brain barrier integrity and reducing malignant edema and hemorrhagic transformation. Although the prematurely terminated CHARM trial was neutral overall, subgroups with moderately large core and those undergoing endovascular therapy suggested functional outcome benefits with glibenclamide in post-hoc sub-analyses [44]. Further development of these and other agents would be of particular benefit in patients presenting to non-thrombectomy centers.

Lastly, improving systems of care to accelerate reperfusion will ultimately benefit all stroke patients, but particularly the fast-progressing patients with large ischemic core. The evidence for EVT benefit in this broader range of patients, including those with large core at a primary stroke center who were then transferred for endovascular therapy, further reduces the indication for repeat imaging on arrival at the comprehensive center, given the delay this incurs.

Endovascular thrombectomy when compared to medical management has been shown to be effective in improving functional outcomes without significant risk of important safety outcomes such as symptomatic intracerebral hemorrhage, decompressive hemicraniectomy and mortality. There is no simple upper limit of core volume beyond which patients do not benefit and a default position to treat gives patients the best chance of minimizing disability. However, in some individuals, the combination of a large core in eloquent locations and premorbid factors that limit the patient's capacity to engage in intensive rehabilitation, mean that the probability of recovery to a level likely to be deemed acceptable by the patient may be regarded as insufficient to warrant treatment. The application of these trial results in the real world setting can be complex, with factors such as age, frailty, time from symptom onset, site of occlusion, severity of stroke and pre-existing patient wishes, all important considerations. A number of adjunctive techniques to improve outcomes in this patient population are being explored including those targeting microvascular hypoperfusion and neuroprotectants, while ultimately systems of care need to be established to ensure equitable access to thrombectomy for this indication.

## Author contributions

Dr Mutimer and Dr Campbell wrote the manuscript.

## Declaration of competing interest

The authors declare that they have no known competing financial interests or personal relationships that could have appeared to influence the work reported in this paper.
